# Recent Developments of the Solution-Processable and Highly Conductive Polyaniline Composites for Optical and Electrochemical Applications

**DOI:** 10.3390/polym11121965

**Published:** 2019-11-29

**Authors:** Sunghun Cho, Jun Seop Lee, Hyeonseo Joo

**Affiliations:** 1 School of Chemical Engineering, Yeungnam University, Gyeongsan 38541, Korea; hyeonssjoo@gmail.com; 2 Department of Materials Science and Engineering, College of Engineering, Gachon University, Seongnam 13120, Korea

**Keywords:** conducting polymer, polyaniline, solution process, secondary doping, thin film electrode

## Abstract

Solution-processable conducting polymers (CPs) are an effective means for producing thin-film electrodes with tunable thickness, and excellent electrical, electrochemical, and optical properties. Especially, solution-processable polyaniline (PANI) composites have drawn a great deal of interest due to of their ease of film-forming, high conductivity up to 10^3^ S/cm, excellent redox behaviors, processability, and scalability. In this review, basic principles, fabrication methods, and applications of solution-processable PANI composites will be discussed. In addition, recent researches on the PANI-based electrodes for solar cells (SCs), electrochromic (EC) windows, thermoelectric (TE) materials, supercapacitors, sensors, antennas, electromagnetic interference (EMI) shielding, organic field-effect transistors (OFETs), and anti-corrosion coatings will be discussed. The presented examples in this review will offer new insights in the design and fabrication of high-performance electrodes from the PANI composite solutions for the development of thin-film electrodes for state-of-art applications.

## 1. Introduction

Conducting polymers (CPs), such as polyaniline (PANI), polyethylene(3,4-dioxythiophene) (PEDOT), and polypyrrole (PPy), are defined as polymeric materials that can provide electrical conductivity after appropriate doping processes, in addition to inherent advantages of polymeric materials, such as processability, light weight, and low cost [1,2,3,4]. In addition, such CPs can be easily polymerized by chemical oxidative polymerization and provide tunable redox properties and good processability from solution [5,6,7,8,9,10,11,12,13,14,15,16,17,18,19,20,21,22,23,24,25,26,27]. Solution-processable CPs are defined as CPs that can form stable dispersion with solvents through intermolecular interactions between the solutes and solvents [5,6,7,8,9,10,11,12,13,14,15,16,17,18,19,20,21,22,23,24,25,26,27]. The CP chain must be prepared in the form of protonated state with two radical cations per one repeating unit in order to make CP chains electrically conductive [1,2,3,4]. The ion-dipole, dipole-dipole, and hydrogen bonding interactions between the protonated CP and the solvent molecules promote the dispersion of the CP chain in the solvent. Therefore, the protonated structures of CP chains can be dissolved in polar solvents, such as *N,N*-dimethyl formamide (DMF), *N*-Methyl-2-pyrrolidone (NMP), *m*-cresol, acetone, ethanol, methanol, water, and so forth [5,6,7,8,9,10,11,12,13,14,15,16,17,18,19,20,21,22,23,24,25,26,27]. Although the protonated portions of CP chains can be dissolved in water, the overall solubility in water is limited due to the non-protonated portions of CP chains being hydrophobic [1,2,3,4,5,6,7,8,9,10,11,12,13,14,15,16,17,18,19,20,21,22,23,24,25,26,27]. The CP chains were combined with hydrophilic polymers, such as poly(4-styrenesulfonate) (PSS) [18,19,20,21,22,23,24,25,26,27,28,29,30,31,32,33,34,35,36,37,38,39,40], carboxymethylcellulose (CMC) [41,42,43,44,45], styrene-butadiene rubber (SBR) [43,44,45], polyacrylic acid (PAA) [46,47,48], polyethylene glycol (PEG) [47], polyethylene oxide (PEO) [49,50], poly(vinyl pyrrolidone) (PVP) [51], and polyvinlyl alcohol (PVA), to enhance the solubility of protonated CPs in water [52]. Water-soluble CP solutions, such as PEDOT:PSS [18,19,20,21], PANI:PSS [22,23,24,25,26,27,28,29,30,31,32,33,34,35,36,37], PPy:PSS [38,39,40], PANI:CMC [41,42,44,45], PANI:SBR [43,44,45], PANI:PAA [46,47,48], PANI:PEG [47], PANI:PEO [49,50,51], PANI:PVP [52], and PANI:PVA [53], provide an efficient way for producing thin-film electrodes having high conductivity, tunable thickness, and good optical transparency. Moreover, the water-based CPs provides better eco-friendliness and less toxicity when compared with the organic solvent-based CPs [22,23,24,25,26,27,28,29,30,31,32,33,34,35,36,37,38,39,40,41,42,43,44,45,46,47,48,49,50,51,52,53]. Among these candidates, the PEDOT:PSS is known as the most commercially available CP, due to its small band gap (1.6−1.7 eV), good optical properties, and high conductivity up to 10^3^ S/cm [18,19,20,21]. However, despite its high conductivity, PEDOT:PSS generally has poor redox properties, which results in lower electrochemical performance than PANI and PPy [18,19,20,21].

PANI solutions, such as polyaniline camphorsulfonic acid (PANI:CSA) and water-soluble PANI, provide superior redox properties, lower cost, and comparable or better conductivity when compared to PEDOT:PSS [5,6,7,8,9,10,11,12,13,14,15,16,17,18,19,20,21,22,23,24,25,26,27,28,29,30,31,32,33,34,35,36,37,41,42,43,44,45,46,47,48,49,50,51,52,53]. In particular, the electrodes obtained from the PANI:CSA solution boast two to three times greater conductivity as compared to conventional PANI electrodes [5,6,7,8,9,10,11,12,13,14,15,16,17]. In addition, the PANI and its composites provide excellent compatibility with various other components, such as inorganics [30,33,34,35,36,37,42,54,55,56,57], carbon nanodots (CNDs) [17], carbon nanotubes (CNTs) [58,59,60], graphene sheets (GSs) [6,31,32,37,41,45,46,61,62,63,64], graphite nanofibers (GNFs) [65], carbon nanoparticles (CNPs) [66], and CPs [20,67]. Therefore, the solution-processable PANI composites have been widely used as electrode materials for solar cells (SCs) [12,13,14,15,17,18,19,25,26,27,47,49,50,51,52,57,64], electrochromic (EC) windows [28,29,30], thermoelectric (TE) materials [20,54,55,58,59,61,68,69], supercapacitors [16,31,32,33,34,45,46,53,60,62,67], sensors [35,36,48,56,65,70], antennas [37,63,66], electromagnetic interference (EMI) shielding [71], organic field-effect transistors (OFETs) [50,72], and anti-corrosion coatings [43,73]. A systematic review will be helpful in understanding the recent progresses and future prospects of solution-processable PANI composites with the rapid development of CP-based electrode materials.

Herein, we report the recent development of solution-processable PANI composites on the efficient fabrication, and its applications as high-performance electrode materials. Additionally, the prospects and technical challenges of fabricating solution-processable PANI composites with higher conductivity, larger surface area, and improved electrochemical performance are discussed. This review is expected to provide detailed information regarding solution-processable PANI composites via various techniques as important alternatives to the expensive PEDOT:PSS.

## 2. Solution-Processable PANI Derived from PANI:CSA

### 2.1. Principle of Secondary Doping of Aniline

**Figure 1:**
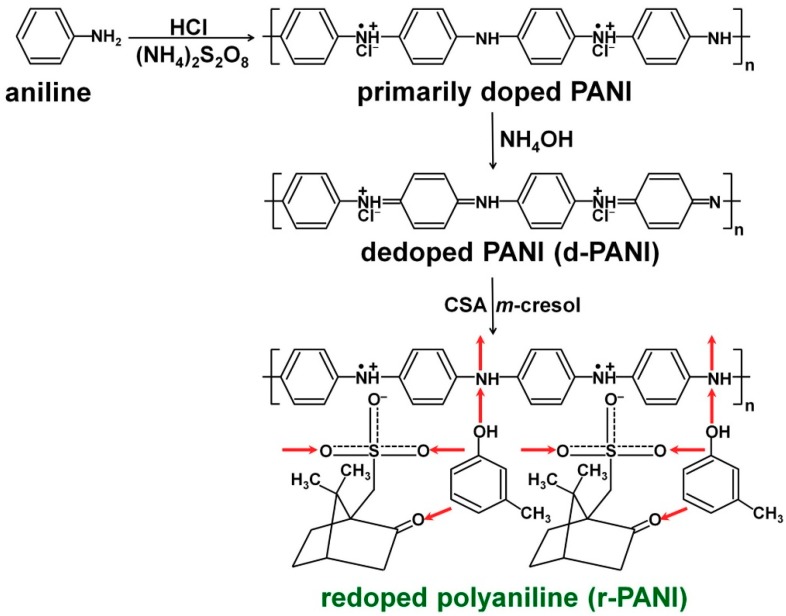
Overall procedures for fabricating polyaniline camphorsulfonic acid (PANI:CSA) while using the secondary doping method. Reprinted with permission from [6]. Copyright 2015, American Chemical Society.

In the first step, chemical oxidative polymerization of the aniline monomer is generally carried out while using hydrochloric acid (HCl) as a dopant and ammonium sulfate (APS) as an oxidant [1,2]. This chemical oxidative polymerization results in a half-oxidized PANI emeraldine salt (ES) form [1,2]. The PANI ES form is converted into the PANI emeraldine base (EB) form by adding reducing agents, such as ammonium hydroxide (NH_4_OH) [5,6,7,8,9,10,11,12,13,14,15,16,17,18,19,20,21]. These doping/dedoping processes can be reversibly carried out. In 1994, MacDiarmid and co-workers proposed a secondary doping mechanism of PANI while using organic acids, such as dodecyl benzene sulfonic acid (DBSA) and camphorsulfonic acid (CSA) [5]. In the secondary doping process, the dedoped PANI EB is redoped by DBSA or CSA. These DBSA and CSA contain big counter ions, and these bulky counter ions extend the distance between the protonated N−H^+^∙ groups of the PANI molecules [5,6]. As the distance between the cations of PANI:DBSA and PANI:CSA increases, the structures of the PANI change from small coils to expanded coils [5,6]. The expanded coil structures promote electron delocalization within the PANI:DBSA and PANI:CSA, which result in improved conductivity when compared to primary-doped PANI structures (Figure 1) [5,6]. In particular, the coil expansion effect is known to be greater in secondary doped PANI with CSA than the secondary doped PANI with DBSA due to the molecular structure of the CSA [5,6]. Thus, CSA is an effective secondary dopant for improving the intermolecular and intermolecular components of the conductivity of PANI. The PANI:CSA offers strong hydrogen bonding interactions with the −SO_3_H group of CSA and −OH group of *m*-cresol, which enables excellent compatibility with the *m*-cresol [5,6,15,16]. In addition, the C−H bonds of chloroform are advantageous for forming dispersion forces with C−H bonds of phenylene rings within the PANI molecules. Therefore, the PANI:CSA can have good compatibility with the *m*-cresol/chloroform *co*-solvent system [5,6,15,16]. Although hydrogen bonding interaction between the PANI:CSA and water molecule is also possible, the solubility of the PANI:CSA in water is poor as compared with the *m*-cresol/chloroform *co*-solvent [5,6,7,8,9,10,11,12,13,14,15,16,17]. Electrodes that are generated from the PANI:CSA solution boast two or three orders of magnitude greater conductivity when compared to conventional PANI electrodes [5,6,7,8,9,10,11,12,13,14,15,16,17,18,19,20]. Moreover, the PANI:CSA was also effective to enhance electrical conductivity within the *isotactic*-polypropylene (*i*-PP) composites [6].

Recently, there have been various approaches for enhancing the electrical conductivity and electrochemical properties of the PANI:CSA for use in various applications, such as SCs [12,13,14,15,17,18,19], TE materials [20,54,55,58,59,61,68,69], supercapacitors [16,60,62,67], chemical sensors [56,65,70], antennas [63,66], EMI shielding [71], OFETs [72], anti-corrosion coatings [73], and so forth.

### 2.2. Conductivity Enhancement of PANI:CSA

**Figure 2:**
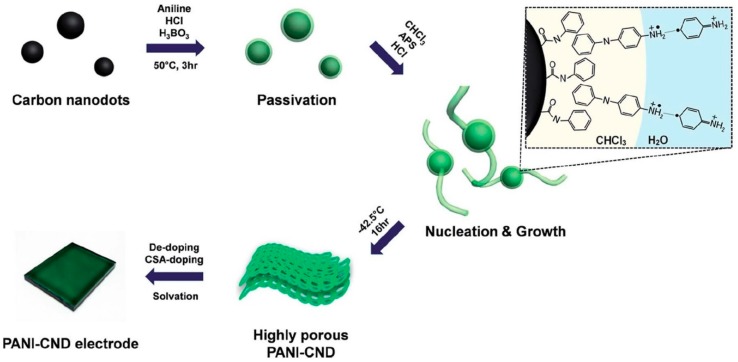
A schematic illustration showing synthesis of PANI:CSA nucleated on carbon nanodots (CNDs) via self-stabilized dispersion polymerization. Reprinted with permission from [17]. Copyright 2015, RSC Publishing.

In the past, studies on improving the conductivity of PANI:CSA have been carried out by controlling the solvent [5,10], temperature [7,11], film-forming time [11], and film-thickness [12]. It was evident that the optimum electrical conductivity was achieved when using a solvent consisting of a higher amount of *m*-cresol and an appropriate amount of CHCl_3_ [5,6,7,8,9,10,11,12,13,14,15,16,17,18,19,20]. According to the method of Kaner et al., more uniform PANI nanofibers (NFs) were readily formed at a water/CHCl_3_ interface when compared to the conventional synthesis of aniline while using a single aqueous phase [2]. In addition, performing the interfacial polymerization at less than −30 °C promotes *para*-coupling of aniline monomers, which resulted in PANI nanostructures with fewer structural defects [7,8]. The secondarily doped of the PANI prepared by the interfacial polymerization has shown the maximum conductivity close to 10^3^ S/cm, which is approximately six times higher than the conventional PANI:CSA synthesized by conventional single-phase polymerization, due to the enhanced crystallinity [9]. Furthermore, Lee et al. reported that the PANI chains can be better aligned through the thickness-controlled drop-casting (TCDC) method [12]. The structural defects in the PANI:CSA were even reduced by using CNDs as nucleating agents during the polymerization of aniline (Figure 2) [17]. Furthermore, PANI:CSA that was grown on the CNDs provides greater surface areas when compared to the conventional PANI:CSA, which results in improved device performances [17]. Tremendous efforts have been made to utilize the PANI:CSA as electrode materials in various devices because of the excellent electrical conductivity of PANI:CSA.

### 2.3. Applications of PANI:CSA for Optical and Electrochemical Devices

#### 2.3.1. PANI:CSA for Solar Cell Application

PANI:CSA has been used as electrode materials for dye-sensitized solar cells (DSSCs) [13,14,15,17], organic solar cells (OSCs) [12,18], and perovskite solar cells (PSCs) [19]. In particular, the PANI:CSA has been studied as counter electrodes (CEs) in the DSSCs [13,14,15,17]. The PANI:CSA is considered to be one of the promising substitutes for platinum-coated transparent conductive oxides (Pt-coated TCOs). This is because the Pt-coated TCOs suffer from mechanical brittleness of TCOs, and it is difficult to cover the large areas of TCOs while using the Pt-sputtering process [14,15]. The PANI:CSA CE has demonstrated a transmittance of 72.9% at 600 nm, and the PCE of DSSCs while using the PANI:CSA CEs was changed by different protonation levels of the PANI structure [13]. Furthermore, the PANI:CSA can be used as a CE in a bifacial DSSC [14]. The PANI:CSA CEs with controllable sizes and shapes of pores could be realized by using different porogens, which results in the relative efficiency of 101.0% (PCE of 6.23%) when compared to the conventional Pt-coated TCO cell (PCE of 6.17%) [15]. Although the thermal decomposition of porogens within the PANI:CSA were helpful in increasing the surface areas of CEs, it was inevitable to avoid undesirable conductivity losses due to the increased surface roughness of the PANI:CSA film [15,16]. After applying the PANI:CSA that was grown on the CNDs as a CE, larger surface areas (43.6 m^2^ g^−1^), higher electrical conductivity (774 S/cm), and superior PCE (7.45%) of the DSSC could be simultaneously achieved [17].

**Figure 3:**
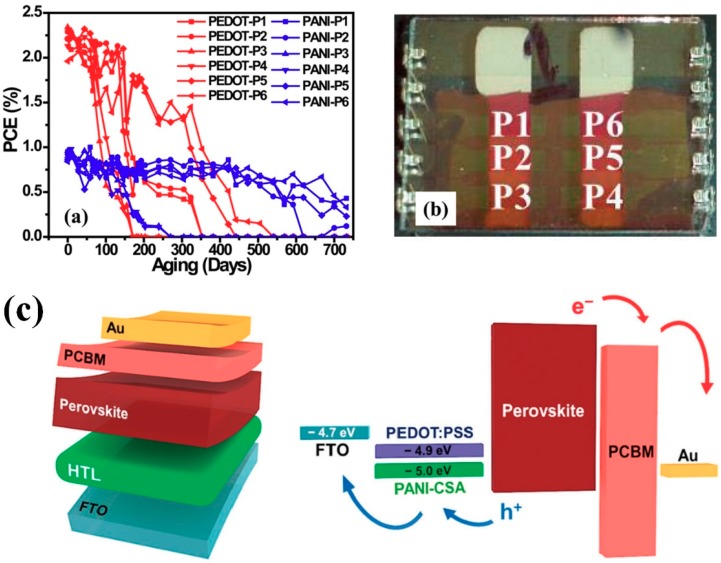
(**a**) Power conversion efficiency (PCE) for pixels of PEDOT:PSS (red) and PANI:CSA (blue) as a function of aging time. (**b**) A photograph of the cell with the designated pixels. Reprinted with permission from [18]. Copyright 2015, American Chemical Society. (**c**) Schematic illustration of the perovskite solar cell (PSC) architecture. Reprinted with permission from [19]. Copyright 2018, RSC Publishing.

Lee et al. demonstrated the OSC while using the PANI:CSA layer and the PEDOT:PSS layer as an anode and a buffer layer, respectively [12]. In the comparative studies on the OSCs using PANI:CSA and PEDOT:PSS as HTLs, the OSC cell that was based on the PANI:CSA exhibited a superior stability as compared to that of the PEDOT:PSS cell (Figure 3a) [18]. PANI:CSA was introduced as a hole transport layer (HTL) to promote hole extraction ability and improve the efficiency and stability of PSCs, and the PANI:CSA exhibited higher power conversion efficiency (PCE) of 15.42% when compared to the PEDOT:PSS with PCE of 14.11% (Figure 3b) [19]. These results indicate that the PANI:CSA can be a promising candidate for HTLs in both PSC and OSC [18,19].

#### 2.3.2. PANI:CSA for PANI:CSA for TE Application

TE performance of the materials is estimated by the TE figure of merit, *ZT*=(*S*^2^·*σ*)*T*/*κ*, where *S*, *σ*, *S*^2^·*σ*, T, and *κ* refer to the Seebeck coefficient, electrical conductivity, power factor (PF), absolute temperature, and thermal conductivity, respectively [68,69]. PANI:CSA with high electrical conductivity (*σ*=10^2^−10^3^ W·m^−1^·K^−1^) and low thermal conductivity (*κ*=0.2−0.4 W·m^−1^·K^−1^) has attracted great attention as a TE material, according to the figure of merit equation [20,54,55,58,59,61,68,69]. Such low *κ* of PANI:CSA is advantageous in lowering the high *κ* values of inorganics and carbons [54,55,58,59,61]. However, the PF (*S*^2^·*σ* =1 μW·m^−1^·K^−2^) of PANI:CSA is about two or three orders of magnitude lower than state-of-art semiconductors based on inorganics and carbons.

**Figure 4:**
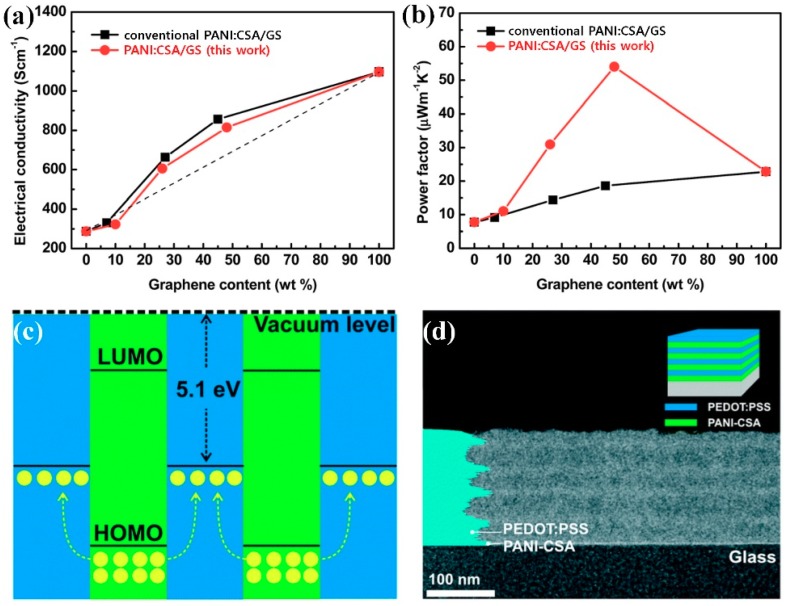
(**a**) The electrical conductivity and (**b**) the power factor of conventional PANI/GS and PANI/GS (this work) composite films at room temperature with different GS contents. Reprinted with permission from [61]. Copyright 2015, RSC Publishing. (**c**) Schematic energy level diagram illustrating the charge transfer process in the PEDOT:PSS/PANI:CSA multilayer structure (the yellow dots represent holes) and (**d**) A transmission electron microscopy cross-sectional image of 5(PEDOT:PSS/PANI–CSA) multilayer films. Reprinted with permission from [20]. Copyright 2016, RSC Publishing.

The TE performances of PANI:CSA were improved by combining the advantages of PANI:CSA with inorganics and carbons [54,55,58,59,61]. The maximum PF (μW·m^−1^·K^−2^) of the PANI:CSA combined with tellurium (Te), CNT, and GS were 146, 401, and 55, respectively (Figure 4a,b) [54,58,61]. Especially, the high TE performances of PANI:CSA/CNT and PANI:CSA/GS are attributable to following reasons [58,59,61]. 1) The phonon scattering at the contact surfaces between PANI and carbons significantly reduces the thermal conductivity of the composites. 2) The high electrical conductivity of carbons and PANI:CSA results in improved TE performances since the mobility of charge carriers in PANI:CSA is still maintained. 3) Synergistic effects between *m*-cresol solvent and carbons also contributes to the formation of highly ordered PANI:CSA chains in the TE device. Interestingly, multilayer structures that were composed of PANI:CSA and PEDOT:PSS were prepared while using a layer-by-layer deposition (Figure 4c,d) [20]. Hole diffusion from the PANI:CSA to the PEDOT:PSS resulted in the maximum PF of 49 μW·m^−1^·K^−2^ [20]. These results suggest that the PANI:CSA is one of fascinating candidates for the TE generators.

#### 2.3.3. Supercapacitor Application

As PANI provides relatively higher electrochemical and pseudocapacitance when compared to the PEDOT and PPy, PANI-based electrodes have attracted a great deal of interest for use in energy storage applications [16,60,62,67]. PANI:CSA significantly improves current collections in the supercapacitors due to its two or three orders of magnitude greater conductivity. Especially, the PANI:CSA electrode can act as a free-standing electrode without using any metallic substrate, such as stainless steel, gold (Au), platinum (Pt), and so forth [16,60,62,67]. However, the low surface area of PANI:CSA films limits the overall performance of supercapacitors [16,60,62,67]. Thus, various efforts have been made to increase the surface area of PANI:CSA electrodes.

A method for incorporating the porous structures into the solution-processable CSA-doped films was conducted to increase the surface area of PANI:CSA electrodes [16]. Porous PANI:CSA electrodes were used working electrodes (WEs) in three-electrode capacitors, and pores of different sizes and shapes formed on the surface of PANI:CSA were effective in enhancing the contact between the PANI:CSA electrodes and the electrolyte ions. However, the capacitance losses that were caused by swelling and degradation of the PANI:CSA films during a number of charge/discharging processes were inevitable. According to Kim et al., the synergistic effects of PANI:CSA and reduced graphene oxide (RGO) were effective in improving the overall performance of supercapacitors [62]. Unique pseudocapacitive behaviors of the PANI:CSA enhance the total capacitance of energy storage devices, while the electron-rich RGO sheets enable significantly improved cycling stability, enlarged surface area, and the higher electric conductivity of electrode materials.

**Figure 5:**
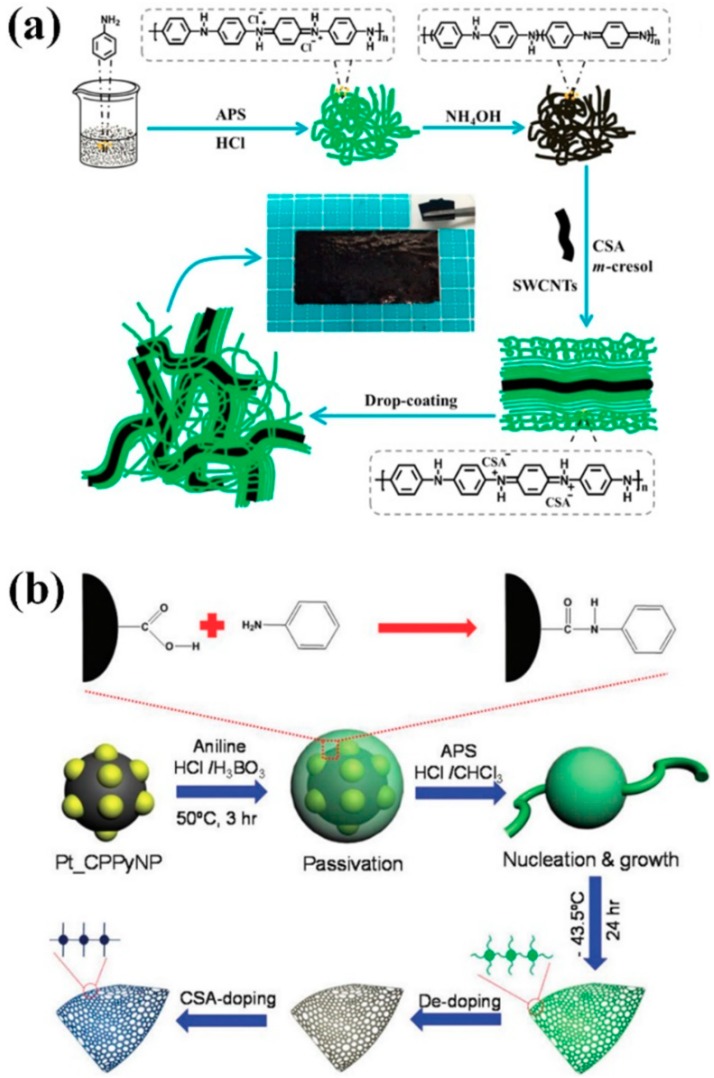
(**a**) Schematic illustration of the fabrication process of PANI:CSA/SWCNT free-standing film. Reprinted with permission from [60]. Copyright 2017, American Chemical Society. (**b**) Illustrative diagram of the fabrication sequence of Pt-CPPy NP embedded PANI:CSA (Pt-CPPy/PANI:CSA) paste. Reprinted with permission from [67]. Copyright 2019, RSC Publishing.

The cycling stability of PANI:CSA was significantly improved by applying the PANI:CSA electrode in a two-electrode configuration [62]. Nanomaterials, such as CNTs and Pt-decorated carboxyl polypyrrole nanoparticles (Pt-CPPy NPs), were combined with the PANI:CSA, and these composite electrodes were used as two-electrode cells [60,67]. It was found that the combination of PANI:CSA with CNT was effective to realize a flexible integrated electrode for a symmetric supercapacitor [60]. Conductive pathways for delocalizing electrons were readily formed in the PANI:CSA through strong π−π interactions between PANI:CSA and CNT. Relatively high gravimetric capacitance and excellent retention rate of 98% after 13000 cycles due to the synergistic effects between PANI:CSA and CNT (Figure 5a) [60]. Interestingly, Pt-CPPy NPs were used as nucleating agents to induce *para*-polymerization of aniline, and the secondary doping process of the PANI grown on Pt-CPPy NPs could significantly improve the electrical conductivity (Figure 5b) [67]. The composite material that was composed of PANI:CSA and Pt-CPPy was used as a two-electrode symmetric supercapacitor, and exhibited significantly improved electrical conductivity (814 S cm^−1^), specific capacitance (325.0 F g^−1^), and cyclic stability (84% of retention rate after 5000 cycles) when compared to conventional PANI:CSA [67]. Despite such improvements, the potential window of symmetric cells based on PANI:CSA is usually less than 1.2V [60,67]. This problem can be solved by choosing an asymmetric cell or non-aqueous electrolyte.

#### 2.3.4. PANI:CSA for Other Applications: Chemical Sensor, Antenna, EMI Shielding, OFET, and Anti-Corrosion

**Figure 6:**
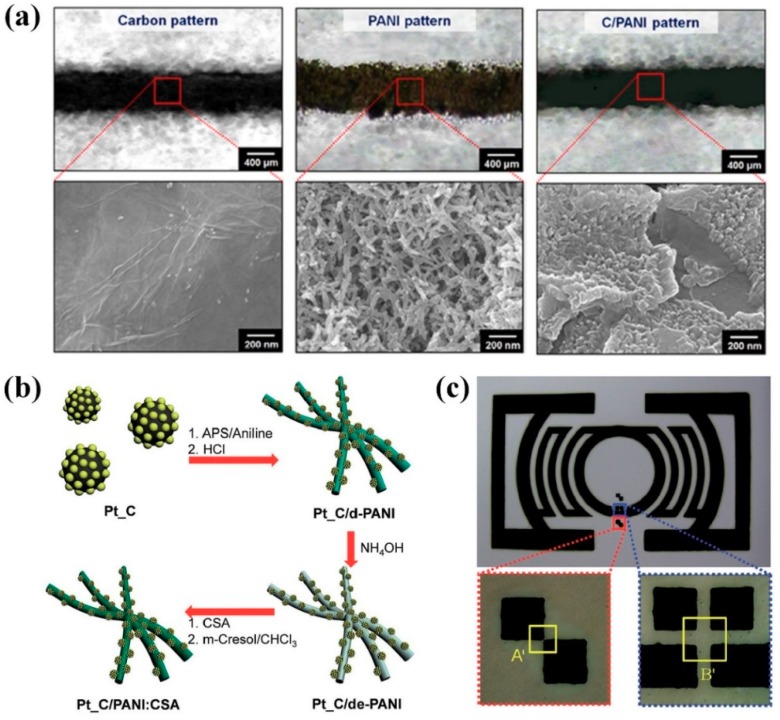
(**a**) Fabrication of conductive films on flexible substrates via screen-printing and a top-view schematic of various two-dimensional (2D) monopole antennas with an SMA (SubMiniature version A) connector. Optical and FE−SEM micrographs show the surface morphology of carbon, PANI, and C/PANI-based straight lines (ca. 500 μm × ca. 30 mm). Reprinted with permission from [63]. Copyright 2015, Nature Publishing Group. (**b**) Illustrative diagram of the sequential steps for Pt-CNP embedded PANI:CSA (Pt-CNP/PANI:CSA) paste and (c) the designed pattern for the dipole tag-antenna and optical images of the enlarged section formed by the Pt-CNP/PANI:CSA paste (the distance along the diagonal direction for the A’ section and the horizontal direction for the B’ section was 50 and 100 mm (r = 5.5 mm, R = 7 mm), respectively). Reprinted with permission from [66]. Copyright 2015, RSC Publishing.

Rapid and reversible doping/dedoping processes make PANI:CSA suitable as efficient sensors in detecting ammonia (NH_3_) gases [56,65,70]. When the PANI:CSA were combined with tin (II) oxide nanoparticles (SnO_2_ NPs) and GNFs, the composite electrodes demonstrated higher sensitivity, faster response, and better selectivity toward NH_3_ when compared with the pure PANI:CSA [56,65]. Furthermore, the SnO_2_ NPs and GNFs could greatly improve the lifetime and structural stability of the PANI:CSA. The PANI:CSA/SnO_2_ composite exhibited superior sensing performances when compared with the pristine PANI and pristine SnO_2_ NPs due to the synergistic effects of the PANI:CSA and SnO_2_ NPs [56]. The PANI:CSA combined with carbon nanomaterials provides higher electrical conductivity and improved flexibility compared to pristine PANI:CSA due to the π−π interactions between the PANI:CSA chains and carbon nanomaterials [6,17,20,58,59,60,61,62,63,65,66]. These advantages of PANI:CSA/carbon composites are suitable for antenna applications. In addition, these PANI:CSA/carbon composites can be readily formed into various patterns with different sizes and shapes while using screen-printing technique [63,66]. The PANI:CSA/GS composite was applied to a monopole antenna, and peak gain, directivity, and radiation efficiency of the monopole antenna based on PANI:CSA/GS were 3.60 dBi, 3.91 dBi, and 92.12%, respectively (Figure 6a) [63]. PANI:CSA NFs that were embedded with Pt-coated carbon nanoparticles (Pt-CNPs) exhibited about 1.37 times higher electrical conductivity (792 S cm^−1^) than that of pristine PANI:CSA NFs (580 S cm^−1^) (Figure 6b,c) [66]. The PANI:CSA/Pt-CNP composite was applied to a dipole tag-antenna that displayed a wide bandwidth of 0.55 GHz and a transmitted power efficiency of 99.6% [66].

**Figure 7:**
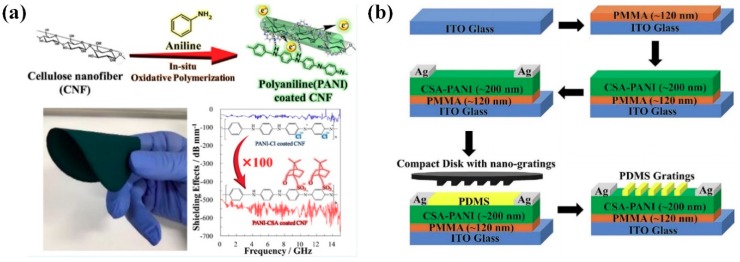
(**a**) Overall processes of fabricating and applying organic thin paper of CNF/PANI:CSA for EMI Shielding. Reprinted with permission from [71]. Copyright 2019, American Chemical Society. (**b**) Process flow used for fabrication of Nano-Grating PANI based Field Effect Transistor. Reprinted with permission from [72]. Copyright 2018, Elsevier.

According to Omura et al., cellulose nanofibers (CNFs) that were coated with the PANI:CSA have shown 193 times higher electrical conductivity (38.5 S cm^−1^) as compared to CNFs coated with HCl-doped PANI (0.20 S cm^−1^) [71]. The CNFs/ PANI:CSA composite electrode demonstrated significantly improved EMI shielding efficiency of −30 dB (−545 dB mm^−1^) in the frequency region from 0.45 to 15 GHz due to its superior electrical conductivity (Figure 7a) [71]. According to Sharma et al., PANI:CSA serves as a conductive layer for enhancing the photoconductivity of an OFET device composed of Ag/PANI:CSA/PMMA/ITO multilayers (Figure 7b) [72]. The saturated hole mobility, threshold voltage, external quantum efficiency, photo-sensitivity, and photo-responsivity of OFET device were 9.5 × 10^−5^, cm^2^/V·s, −1.72 V, 1.16 × 10^2^ A/W, and 7.33 × 10^4^ A/W, respectively [72]. Furthermore, the PANI:CSA/SiO_2_ core-shell can be used for the corrosion protection coating for carbon steel substrates [45]. The PANI:CSA/SiO_2_ core-shell has shown nearly five orders magnitude higher corrosion resistance (2.24 × 10^7^ Ω cm^2^) when compared with the pristine silicon coating (5.37 × 10^2^ Ω cm^2^) [73]. This result is indicative of the improved physical barrier behavior of the PANI:CSA.

## 3. Solution-Processable PANI Derived from Water-Based Systems

### 3.1. Water-Soluble PANI:PSS for Optical and Electrochemical Applications

**Figure 8:**
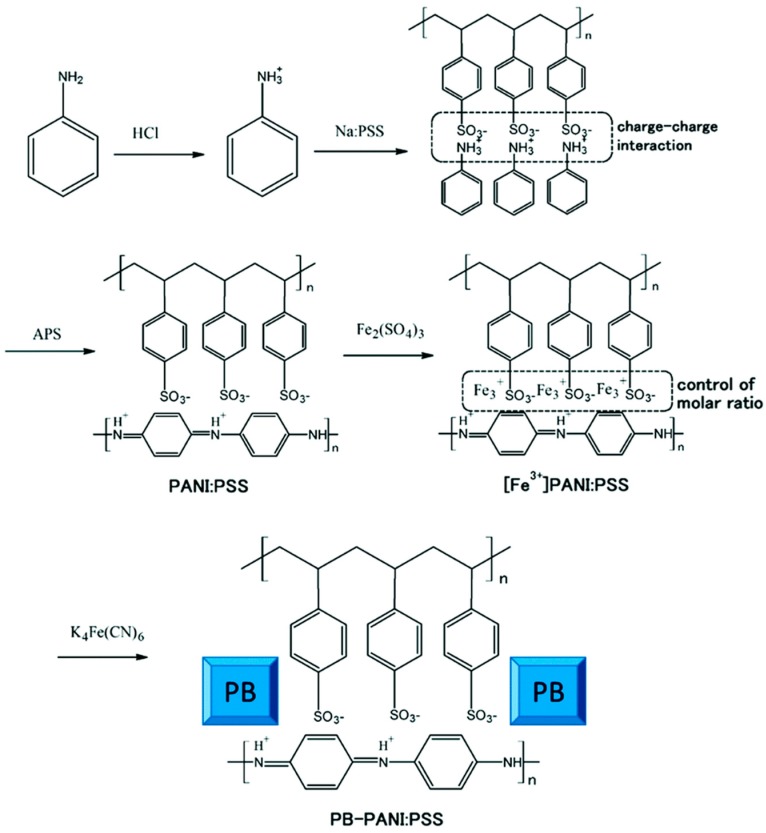
Synthesis route for the preparation of the Prussian blue–PANI:PSS (PB–PANI:PSS) composite. Reprinted with permission from [28]. Copyright 2016, RSC Publishing.

The PSS, a vinyl polymer having sulfonate (−SO_3_^−^) groups promotes para-directed polymerization of aniline, which resulted in the formation of PANI with lower structural defects [18,19,20,21,22,23,24,25,26,27,28,29,30,31,32,33,34,35,36,37,38,39,40]. A water-soluble PANI:PSS was proposed as an alternative to substitute PEDOT:PSS with high cost and poor redox behaviors [22,23,24,25,26,27,28,29,30,31,32,33,34,35,36,37]. The synthesis of PANI:PSS NPs was proposed by simple chemical oxidative polymerization of aniline in the presence of the PSS (Figure 8) [24,25,26,27,28].

**Figure 9:**
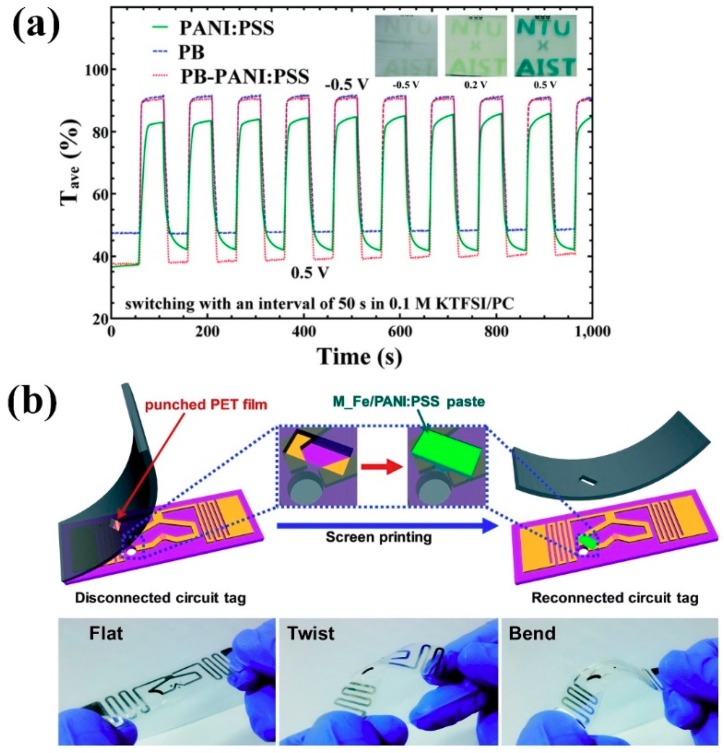
(**a**) Tave of PANI:PSS, PB, and PB–PANI:PSS thin films switched between −0.5 and +0.5 V with an interval of 50 s. Reprinted with permission from [28]. Copyright 2016, RSC Publishing. (**b**) Schematic diagram of the sequential fabrication process of the RFID sensor tag and photographs of the radio frequency identification (RFID)-tag sensor under various deformations. Reprinted with permission from [36]. Copyright 2019, RSC Publishing.

The sizes and electrical conductivities of PANI:PSS are dependent on the molecular weight (Mw) of PSS, which results in a different sensitivity toward H_2_S molecules [22]. The electrostatic interaction between the protonated PANI chain and negatively charged PSS chain form a water-soluble copolymer [22,23,24]. Furthermore, the −SO_3_^−^ groups of PANI:PSS enable electrostatic interactions with various materials, such as carbons, inorganics, and polymers [20,21,22,30,31,32,33,34,35,36,37]. Therefore, various PANI:PSS-based composites were designed for SCs [23,25,26,27], ECs [28,29,30], supercapacitors [31,32,33,34], sensors [22,24,35,36], antennas [37], and so forth. It was obvious that the PANI:PSS can act as an efficient hole transport layer (HTL) material in perovskite solar cells (PSCs) and organic solar cells (OSCs) [23,25,26]. A multi-color EC window using the Prussian blue (PB)-PANI:PSS nanocomposite exhibited an average transimittance (ΔT_ave_) of 52.3%, bleaching time of 8.1 s, and darkening time of 13.3 s (Figure 9a) [28]. A photovoltaic-electrochromic (PV-EC) module that was based on PANI:PSS worked by the irradiation of sunlight, and the PV-EC based on PANI:PSS exhibited color changes by tuning the oxidation level of PANI [29]. PANI:PSS/GS composites offer the synergies of psuedocapacitive and electric double-layer (EDLC) mechanisms, which results in improved cycling stability and higher power density [31,32]. Asymmetric supercapacitor based on NiO/PANI:PSS demonstrated a gravimetric capacitance (834 F g^−1^) and a retention rate of 88.9% after 3000 cycles of the charge/discharge processes, which suggests that the introduction of NiO significantly enhanced the energy density of the PANI:PSS [33]. A hybrid paste that was composed of PANI:PSS and platinum-decorated reduced graphene oxide (Pt-RGO) was applied as a micropatterned dipole-tag antenna, and the hybrid paste exhibited an electrical conductivity of 245.3 S cm^−1^ [37]. This indicates that the PANI:PSS-based materials have comparable or similar conductivity with the PEDOT:PSS. NH_3_ could be detectectable at a concentration of 50 percent to parts per trillion (ppt) by using PANI:PSS tubules that were embedded with TiO_2_ NPs [35]. A radio frequency identification (RFID)-based wireless sensor while using the PANI:PSS composite combined with multidimensional Fe_2_O_3_ hollow nanoparticles (M_FeHNPs) was demonstrated to detect NO_2_ at the lowest concentration of 0.5 percent to parts per million (ppm) (Figure 9b) [36]. The application range of PANI:PSS in the field of state-of-art devices will be expanded because of its excellent solution-processability and unique redox behaviors.

### 3.2. Other Water-Soluble PANI Solutions for Optical and Electrochemical Applications

**Figure 10:**
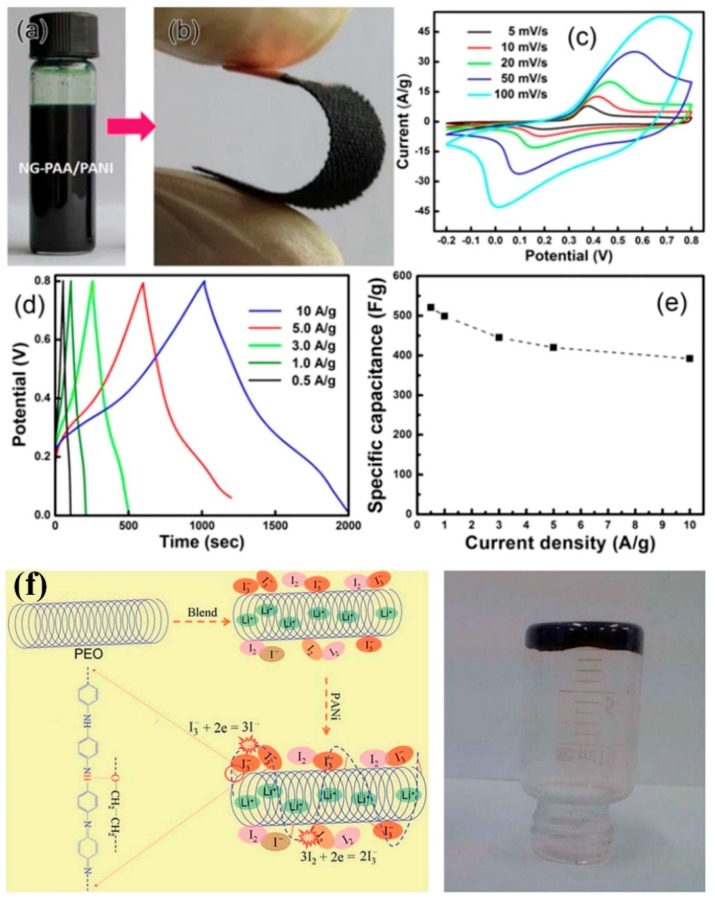
Photographs of (**a**) the aqueous nitrogen-doped graphene-polyacrylic acid/polyaniline (NG-PAA/PANI) suspension containing 32 wt.% PANI and 1.3 wt.% NG, (**b**) a single bent carbon cloth electrode. (**c**) CV curves at different scan rates and (**d**) Galvanostatic charge/discharge curves at different current densities of the optimal NG-PAA/PANI on carbon cloth in 1 M H_2_SO_4_ and (**e**) the corresponding specific capacitance vs. current density. Reprinted with permission from [46]. Copyright 2016, Nature Publishing Group. (**f**) Schematic illustration of the synthesis of (I^−^/I_3_^−^)-incorporated PEO/PANI solid-state electrolytes (left). The PANI chains are bonded onto PEO backbones by H-bonding for catalyzing I_3_^−^ reduction. Digital photograph showing the inverted PEO/1.0 wt% PANI solid electrolyte (right). Reprinted with permission from [49]. Copyright 2015, RSC Publishing.

Other hydrophilic polymers, such as carboxymethylcellulose (CMC) [41,42,44,45], styrene-butadiene rubber (SBR) [43,44,45], polyacrylic acid (PAA) [46,47,48], polyethylene glycol (PEG) [47], polyethylene oxide (PEO) [49,50,51], poly(vinyl pyrrolidone) (PVP) [52], and polyvinlyl alcohol (PVA) [53], and these water-soluble polymers significantly improve the dispersion of PANI in the aqueous phase. In addition, when these water-soluble polymers are used as binders for electrodes or electrolyte membranes, the suppression of undesirable volumetric expansion and durability of the device can be improved [43,44,45,46,47,48,49,50,51,52,53]. According to Bilal et al., the gravimetric capacitance of a three-electrode capacitor while using the GO-PANI/CMC composite was 1721 F g^−1^, which suggested that the dispersion of active materials highly affect the resulting electrochemical performances [41]. Moreover, a mixture of CMC/SBR composite as a water-soluble binder offers the effective suppression of significant volume variations and the interface maintenance of electrode materials, which led to significant improvements in the electrochemical performances of both the Li-ion battery and supercapacitor [44,45]. The solid-state carbon cloth supercacitor based on PANI/CNTs/PAA composites demonstrated an energy density of 5.8 Wh/kg at a power density of 1.1 kW/kg and a rate capability of 81% in the current range from 1 to 10 A/g (Figure 10a−e) [46]. A quasi solid state DSSC (QS-DSSC) was assembled with the PANI/PAA-g-PEG graft composite was used as a gel electrolyte, and the QS-DSSC that was based on the gel electrolyte exhibited a PCE of 6.38% under a solar illumination of 100 mW cm^−2^ (AM 1.5) [47]. In addition, the PANI/PAA film that was immobalized by glucose oxidase (GOx) was effective in detecting glucose molecules, and the sensitivity toward glucose increased with increasing PAA content [48]. The results suggest that the PANI/PAA composites are suitable for fabricating various electrochemical and optical devices [46,47,48]. Furthermore, PANI composites that were combined with PEO and PVP were also utilized as gel electrolytes for offering catalytic and hole-transporting properties on the QS-DSSCs (Figure 10f) [49,50,51,52]. While considering these results, it was evident that the PANI composites combined with water-soluble polymers are appropriate for constructing high-performance and solid-state energy storage and energy conversion devices [43,44,45,46,47,48,49,50,51,52]. According to Li et al., a PANI-PVA hydrogel with a tensile strength of 5.3 MPa can be readily produced by crosslinking reactions between PANI and PVA chains through boronate bonds [53]. The flexible solid-state supercapacitor based on the PANI-PVA hydrogel provided large gravimetric capacitance (928 F g^−1^) and excellent capacitance retention (90% after 1000 charge/discharge cycles) [53].

## 4. Conclusions

In this review, recent researches on solution-processable PANI composites and their applications were discussed. In addition to intrinsic advantages of the PANI, such as facile synthesis, unique redox behavior, reversible doping/dedoping, and low cost, PANI:CSA that was prepared by secondary doping enables facile formation of free-standing thin films with significantly improved electrical and electrochemical performances. The total performances of PANI:CSA could be reinforced by combining it with inorganics, carbons, CPs, and so forth, as the PANI:CSA solution enables hydrogen bonding, dipole-dipole, and ion-dipole forces with various compounds. For this reason, the PANI:CSA and its composites have been widely used in a variety of applications, such as SCs, TE materials, supercapacitors, chemical sensors, antennas, EMI shielding, OFETs, and anti-corrosion coatings. PANI:CSA should overcome several problems, such as difficulty in controlling gelation time and odor characteristic of *m*-cresol solvent, to replace expensive PEDOT:PSS in a wider range of applications. The advantages of the water-soluble PANI composites, such as, low cost, low toxicity, and eco-friendliness, will quickly increase the demand for water-soluble PANI in the fields of high-tech devices. Especially, these water-based PANI composites are highly advantageous for realizing solid-state devices. Developing the fabricating procedures for solution-processable PANI composites will remarkably improve the performances of various state-of-art devices, such as wireless sensors, wireless energy storage/conversion system, smart windows, and so forth.

## References

[1] Wu Y., Wang J., Ou B., Zhao S., Wang Z. (2019). Some Important Issues of the Commercial Production of 1-D Nano-PANI. Polymers.

[2] Huang J., Kaner R.B. (2004). A General Chemical Route to Polyaniline Nanofibers. J. Am. Chem. Soc..

[3] Mantione D., Del Agua I., Sanchez-Sanchez A., Mecerreyes D. (2017). Poly(3,4-ethylenedioxythiophene) (PEDOT) Derivatives: Innovative Conductive Polymers for Bioelectronics. Polymers.

[4] Yan B., Wu Y., Guo L. (2017). Recent Advances on Polypyrrole Electroactuators. Polymers.

[5] MacDiarmid A.G., Epstein A.J. (1995). Secondary doping in polyaniline. Synth. Met..

[6] Cho S., Kim M., Lee J.S., Jang J. (2015). Polypropylene/Polyaniline Nanofiber/Reduced Graphene Oxide Nanocomposite with Enhanced Electrical, Dielectric, and Ferroelectric Properties for a High Energy Density Capacitor. ACS Appl. Mater. Interfaces.

[7] Lee S.-H., Lee D.-H., Lee K., Lee C.-W. (2005). High-Performance Polyaniline Prepared via Polymerization in a Self-Stabilized Dispersion. Adv. Funct. Mater..

[8] Kim M., Cho S., Song J., Son S., Jang J. (2012). Controllable Synthesis of Highly Conductive Polyaniline Coated Silica Nanoparticles Using Self-Stabilized Dispersion Polymerization. ACS Appl. Mater. Interfaces.

[9] Lee K., Cho S., Heum Park S., Heeger A.J., Lee C.-W., Lee S.-H. (2006). Metallic transport in polyaniline. Nature.

[10] Krukiewicz K., Katunin A. (2016). The effect of reaction medium on the conductivity and morphology of polyaniline doped with camphorsulfonic acid. Synth. Met..

[11] Rannou P., Nechtschein M., Travers J.P., Berner D., Woher A., Djurado D. (1999). Ageing of PANI: Chemical, structural and transport consequences. Synth. Met..

[12] Lee B.H., Park S.H., Back H., Lee K. (2011). Novel Film-Casting Method for High-Performance Flexible Polymer Electrodes. Adv. Funct. Mater..

[13] Jeon S.S., Kim C., Lee T.H., Lee Y.W., Do K., Ko J., Im S.S. (2012). Camphorsulfonic Acid-Doped Polyaniline Transparent Counter Electrode for Dye-Sensitized Solar Cells. J. Phys. Chem. C.

[14] Park S.H., Shin K.-H., Kim J.-Y., Yoo S.J., Lee K.J., Shin J., Choi J.W., Jang J., Sung Y.-E. (2012). The application of camphorsulfonic acid doped polyaniline films prepared on TCO-free glass for counter electrode of bifacial dye-sensitized solar cells. J. Photochem. Photobiol..

[15] Cho S., Hwang S.H., Kim C., Jang J. (2012). Polyaniline porous counter-electrodes for high performance dye-sensitized solar cells. J. Mater. Chem..

[16] Cho S., Shin K.-H., Jang J. (2013). Enhanced Electrochemical Performance of Highly Porous Supercapacitor Electrodes Based on Solution Processed Polyaniline Thin Films. ACS Appl. Mater. Interfaces.

[17] Lee K., Cho S., Kim M., Kim J., Ryu J., Shin K.-Y., Jang J. (2015). Highly porous nanostructured polyaniline/carbon nanodots as efficient counter electrodes for Pt-free dye-sensitized solar cells. J. Mater. Chem. A.

[18] Abdulrazzaq O., Bourdo S.E., Woo M., Saini V., Berry B.C., Ghosh A., Biris A.S. (2015). Comparative Aging Study of Organic Solar Cells Utilizing Polyaniline and PEDOT:PSS as Hole Transport Layers. ACS Appl. Mater. Interfaces.

[19] Lee K., Yu H., Lee J.W., Oh J., Bae S., Kim S.K., Jang J. (2018). Efficient and moisture-resistant hole transport layer for inverted perovskite solar cells using solution-processed polyaniline. J. Mater. Chem. C.

[20] Lee H.J., Anoop G., Lee H.J., Kim C., Park J.W., Choi J., Kim H., Kim Y.J., Lee E., Lee S.G. (2016). Enhanced thermoelectric performance of PEDOT:PSS/PANI–CSA polymer multilayer structures. Energy Environ. Sci..

[21] Cho S., Kim M., Jang J. (2015). Screen-Printable and Flexible RuO_2_ Nanoparticle-Decorated PEDOT:PSS/Graphene Nanocomposite with Enhanced Electrical and Electrochemical Performances for High-Capacity Supercapacitor. ACS Appl. Mater. Interfaces.

[22] Cho S., Lee J.S., Jun J., Kim S.G., Jang J. (2014). Fabrication of water-dispersible and highly conductive PSS-doped PANI/graphene nanocomposites using a high-molecular weight PSS dopant and their application in H_2_S detection. Nanoscale.

[23] Lee K., Cho K.H., Ryu J., Yun J., Yu H., Lee J., Na W., Jang J. (2017). Low-cost and efficient perovskite solar cells using a surfactant-modified polyaniline:poly(styrenesulfonate) hole transport material. Electrochim. Acta.

[24] Jang J., Ha J., Cho J. (2007). Fabrication of Water-Dispersible Polyaniline-Poly(4-styrenesulfonate) Nanoparticles for Inkjet-Printed Chemical-Sensor Applications. Adv. Mater..

[25] Yoo J.E., Lee K.S., Garcia A., Tarver J., Gomez E.D., Baldwin K., Sun Y., Meng H., Nguyen T.Q., Loo Y.L. (2010). Directly patternable, highly conducting polymers for broad applications in organic electronics. Proc. Natl. Acad. Sci. USA.

[26] Isakova A., Topham P.D. (2017). Polymer strategies in perovskite solar cells. J. Polym. Sci. Pol. Phys..

[27] Ecker B., Posdorfer J., von Hauff E. (2013). Influence of hole extraction efficiency on the performance and stability of organic solar Cells. Sol. Energy Mater. Sol. Cells.

[28] Hu C.-W., Kawamoto T., Tanaka H., Takahashi A., Lee K.-M., Kao S.-Y., Liao Y.-C., Ho K.-C. (2016). Water processable Prussian blue–polyaniline:polystyrene sulfonate nanocomposite (PB–PANI:PSS) for multi-color electrochromic applications. J. Mater. Chem. C.

[29] Huang L.M., Hu C.W., Peng C.Y., Su C.H., Ho K.C. (2016). Integration of polyelectrolyte based electrochromic material in printable photovoltaic electrochromic module. Sol. Energy Mater. Sol. Cells.

[30] Xiong S., Lan J., Yin S., Wang Y., Kong Z., Gong M., Wu B., Chu J., Wang X., Zhang R. (2018). Enhancing the electrochromic properties of polyaniline via coordinate bond tethering the polyaniline with gold colloids. Sol. Energy Mater. Sol. Cells.

[31] Fenoy G.E., Van der Schueren B., Scotto J., Boulmedais F., Ceolín M.R., Bégin-Colin S., Bégin D., Marmisollé W.A., Azzaroni O. (2018). Layer-by-layer assembly of iron oxide-decorated few-layer graphene/PANI:PSS composite films for high performance supercapacitors operating in neutral aqueous electrolytes. Electrochim. Acta.

[32] Yang C., Zhang L., Hu N., Yang Z., Wei H., Xu Z.J., Wang Y., Zhang Y. (2016). Densely-packed graphene/conducting polymer nanoparticle papers for high-volumetric-performance flexible all-solid-state supercapacitors. Appl. Surf. Sci..

[33] Cho E.-C., Chang-Jian C.-W., Lee K.-C., Huang J.-H., Ho B.-C., Ding Y.-R., Hsiao Y.-S. (2019). Spray-dried nanoporous NiO/PANI:PSS composite microspheres for high-performance asymmetric supercapacitors. Compos. B Eng..

[34] Huang H., Yao J., Chen H., Zeng X., Chen C., She X., Li L. (2016). Facile preparation of halloysite/polyaniline nanocomposites via in situ polymerization and layer-by-layer assembly with good supercapacitor performance. J. Mater. Sci..

[35] Ranka P., Sethi V., Contractor A.Q. (2018). One step electrodeposition of composite of PANI-PSS tubules with TiO_2_ nanoparticles and application as electronic sensor device. Sens. Actuator B Chem..

[36] Kim S.G., Jun J., Lee J.S., Jang J. (2019). A highly sensitive wireless nitrogen dioxide gas sensor based on an organic conductive nanocomposite paste. J. Mater. Chem. A.

[37] Lee J.S., Kim M., Lee C., Cho S., Oh J., Jang J. (2015). Platinum-decorated reduced graphene oxide/polyaniline:poly(4-styrenesulfonate) hybrid paste for flexible dipole tag-antenna applications. Nanoscale.

[38] Han H., Lee J.S., Cho S. (2019). Comparative Studies on Two-Electrode Symmetric Supercapacitors Based on Polypyrrole:Poly(4-styrenesulfonate) with Different Molecular Weights of Poly(4-styrenesulfonate). Polymers.

[39] Kang K.S., Jee C., Bae J.-H., Kim E., Jung H.J., Yang J.Y., Huh P. (2018). Semi-crystalline polypyrrole: Polystyrene sulfonate synthesized through the pores of filter paper. Polym. Eng. Sci..

[40] Pattananuwat P., Tagaya M., Kobayashi T. (2018). Controllable nanoporous fibril-like morphology by layer-by- layer self-assembled films of bioelectronics poly(pyrrole-co-formyl pyrrole)/polystyrene sulfonate for biocompatible electrode. Mater. Res. Bull..

[41] Bilal S., Fahim M., Firdous I., Ali Shah A.-u.-H. (2018). Insight into capacitive performance of polyaniline/graphene oxide composites with ecofriendly binder. Appl. Surf. Sci..

[42] Tanzifi M., Tavakkoli Yaraki M., Karami M., Karimi S., Dehghani Kiadehi A., Karimipour K., Wang S. (2018). Modelling of dye adsorption from aqueous solution on polyaniline/carboxymethyl cellulose/TiO_2_ nanocomposites. J. Colloid Interface Sci..

[43] De León-Almazán C.M., Onofre-Bustamante E., Rivera-Armenta J.L., Ángeles San Martín M.E., Chávez-Cinco M.Y., Gallardo-Rivas N.V., Páramo-García U. (2017). PAni/SBR composite as anticorrosive coating for carbon steel, part II: Electrochemical characterization. Polym. Bull..

[44] Li J.-T., Wu Z.-Y., Lu Y.-Q., Zhou Y., Huang Q.-S., Huang L., Sun S.-G. (2017). Water Soluble Binder, an Electrochemical Performance Booster for Electrode Materials with High Energy Density. Adv. Energ. Mater..

[45] Song B., Wu F., Zhu Y., Hou Z., Moon K.-s., Wong C.-P. (2018). Effect of polymer binders on graphene-based free-standing electrodes for supercapacitors. Electrochim. Acta.

[46] Wang Y., Tang S., Vongehr S., Ali Syed J., Wang X., Meng X. (2016). High-Performance Flexible Solid-State Carbon Cloth Supercapacitors Based on Highly Processible N-Graphene Doped Polyacrylic Acid/Polyaniline Composites. Sci. Rep..

[47] Liu Q., Wu J., Lan Z., Zheng M., Yue G., Lin J., Huang M. (2015). Preparation of PAA-g-PEG/PANI polymer gel electrolyte and its application in quasi solid state dye-sensitized solar cells. Polym. Eng. Sci..

[48] Homma T., Kondo M., Kuwahara T., Shimomura M. (2015). Polyaniline/poly(acrylic acid) composite film: A promising material for enzyme-aided electrochemical sensors. Eur. Polym. J..

[49] Duan Y., Tang Q., Chen Y., Zhao Z., Lv Y., Hou M., Yang P., He B., Yu L. (2015). Solid-state dye-sensitized solar cells from poly(ethylene oxide)/polyaniline electrolytes with catalytic and hole-transporting characteristics. J. Mater. Chem. A.

[50] Rijos L.M., Melendez A., Oyola R., Pinto N.J. (2019). Effect of polyethylene oxide on camphor sulfonic acid doped polyaniline thin film field effect transistor with ionic liquid gating. Synth. Met..

[51] Hsu Y.-C., Tseng L.-C., Lee R.-H. (2014). Graphene oxide sheet–polyaniline nanohybrids for enhanced photovoltaic performance of dye-sensitized solar cells. J. Polym. Sci. Pol. Phys..

[52] Gao J., Yang Y., Zhang Z., Yan J., Lin Z., Guo X. (2016). Bifacial quasi-solid-state dye-sensitized solar cells with Poly(vinyl pyrrolidone)/polyaniline transparent counter electrode. Nano Energy.

[53] Li W., Gao F., Wang X., Zhang N., Ma M. (2016). Strong and Robust Polyaniline-Based Supramolecular Hydrogels for Flexible Supercapacitors. Angew. Chem..

[54] Wang Y., Zhang S.M., Deng Y. (2016). Flexible low-grade energy utilization devices based on high-performance thermoelectric polyaniline/tellurium nanorod hybrid films. J. Mater. Chem. A.

[55] Anno H., Yamaguchi K., Nakabayashi T., Kurokawa H., Akagi F., Hojo M., Toshima N. (2011). Thermoelectric properties of conducting polyaniline/BaTiO_3_ nanoparticle composite films. IOP Conf. Ser. Mater. Sci. Eng..

[56] Khuspe G.D., Navale S.T., Chougule M.A., Patil V.B. (2013). Ammonia gas sensing properties of CSA doped PANi-SnO_2_ nanohybrid thin films. Synth. Met..

[57] Lee R.-H., Chi C.-H., Hsu Y.-C. (2013). Platinum nanoparticle/self-doping polyaniline composite-based counter electrodes for dye-sensitized solar cells. J. Nanopart. Res..

[58] Li H., Liu S., Li P., Yuan D., Zhou X., Sun J., Lu X., He C. (2018). Interfacial control and carrier tuning of carbon nanotube/polyaniline composites for high thermoelectric performance. Carbon.

[59] Yao Q., Wang Q., Wang L., Chen L. (2014). Abnormally enhanced thermoelectric transport properties of SWNT/PANI hybrid films by the strengthened PANI molecular ordering. Energy Environ. Sci..

[60] Liu F., Luo S., Liu D., Chen W., Huang Y., Dong L., Wang L. (2017). Facile Processing of Free-Standing Polyaniline/SWCNT Film as an Integrated Electrode for Flexible Supercapacitor Application. ACS Appl. Mater. Interfaces.

[61] Wang L., Yao Q., Bi H., Huang F., Wang Q., Chen L. (2015). PANI/graphene nanocomposite films with high thermoelectric properties by enhanced molecular ordering. J. Mater. Chem. A.

[62] Kim M., Lee C., Jang J. (2014). Fabrication of Highly Flexible, Scalable, and High-Performance Supercapacitors Using Polyaniline/Reduced Graphene Oxide Film with Enhanced Electrical Conductivity and Crystallinity. Adv. Funct. Mater..

[63] Shin K.-Y., Kim M., Lee J.S., Jang J. (2015). Highly Omnidirectional and Frequency Controllable Carbon/Polyaniline-based 2D and 3D Monopole Antenna. Sci. Rep..

[64] Hsu Y.-C., Chen G.-L., Lee R.-H. (2014). Graphene oxide sheet-polyaniline nanocomposite prepared through in-situ polymerization/deposition method for counter electrode of dye-sensitized solar cell. J. Polym. Res..

[65] Mohammed H.A., Rashid S.A., Abu Bakar M.H., Ahmad Anas S.B., Mahdi M.A., Yaacob M.H. (2019). Fabrication and Characterizations of a Novel Etched-tapered Single Mode Optical Fiber Ammonia Sensors Integrating PANI/GNF Nanocomposite. Sens. Actuator B Chem..

[66] Lee J.S., Kim M., Oh J., Kim J., Cho S., Jun J., Jang J. (2015). Platinum-decorated carbon nanoparticle/polyaniline hybrid paste for flexible wideband dipole tag-antenna application. J. Mater. Chem. A.

[67] Oh J., Kim Y.K., Lee J.S., Jang J. (2019). Highly porous structured polyaniline nanocomposites for scalable and flexible high-performance supercapacitors. Nanoscale.

[68] Yoon C.O., Reghu M., Moses D., Cao Y., Heeger A.J. (1995). Thermoelectric power of doped polyaniline near the metal-insulator transition. Synth. Met..

[69] Anno H., Hokazono M., Akagi F., Hojo M., Toshima N. (2013). Thermoelectric Properties of Polyaniline Films with Different Doping Concentrations of (±)-10-Camphorsulfonic Acid. J. Electron. Mater..

[70] Verma D., Dutta V. (2008). Role of novel microstructure of polyaniline-CSA thin film in ammonia sensing at room temperature. Sens. Actuator B Chem..

[71] Omura T., Chan C.H., Wakisaka M., Nishida H. (2019). Organic Thin Paper of Cellulose Nanofiber/Polyaniline Doped with (±)-10-Camphorsulfonic Acid Nanohybrid and Its Application to Electromagnetic Shielding. ACS Omega.

[72] Sharma S., Khosla R., Das S., Shrimali H., Sharma S.K. (2018). High-performance CSA-PANI based organic phototransistor by elastomer gratings. Org. Electron..

[73] Shi S., Zhao Y., Zhang Z., Yu L. (2019). Corrosion protection of a novel SiO2@PANI coating for Q235 carbon steel. Prog. Org. Coat..

